# Introducing a Novel Experimental Model for Osseo-Disintegration of Titanium Dental Implants Induced by Monobacterial Contamination: An In-Vivo Feasibility Study

**DOI:** 10.3390/ma14227076

**Published:** 2021-11-22

**Authors:** Christian Flörke, Anne-Katrin Eisenbeiß, Ulla Metz, Aydin Gülses, Yahya Acil, Jörg Wiltfang, Hendrik Naujokat

**Affiliations:** Department of Oral and Maxillofacial Surgery, UKSH, Christian-Albrechts-University, 24105 Kiel, Germany; anne-katrin.eisenbeiss@uksh.de (A.-K.E.); ullametzibetzi@gmail.com (U.M.); yahya.acil@uksh.de (Y.A.); joerg.wiltfang@uksh.de (J.W.); hendrik.naujokat@uksh.de (H.N.)

**Keywords:** dental, *Enterococcus faecalis*, implant, osseointegration, peri-implantitis

## Abstract

*Background and Objectives*: The aim of the current study was to establish an osseo-disintegration model initiated with a single microorganism in mini-pigs. *Materials and Methods*: A total of 36 titanium dental implants (3.5 mm in diameter, 9.5 mm in length) was inserted into frontal bone (n: 12) and the basis of the corpus mandible (n: 24). Eighteen implants were contaminated via inoculation of *Enterococcus faecalis*. Six weeks after implant insertion, bone-to-implant contact (BIC) ratio, interthread bone density (ITBD), and peri-implant bone density (PIBD) were examined. In addition to that, new bone formation was assessed via fluorescence microscopy, histomorphometry, and light microscopical examinations. *Results*: Compared to the sterile implants, the contaminated implants showed significantly reduced BIC (*p* < 0.001), ITBD (*p* < 0.001), and PBD (*p* < 0.001) values. Around the sterile implants, the green and red fluorophores were overlapping and surrounding the implant without gaps, indicating healthy bone growth on the implant surface, whereas contaminated implants were surrounded by connective tissue. *Conclusions*: The current experimental model could be a feasible option to realize a significant alteration of dental-implant osseointegration and examine novel surface decontamination techniques without impairing local and systemic inflammatory complications.

## 1. Introduction

Within the last few decades, dental implantology has become a routine treatment option in the daily dental practice. However, parallel to the increasing numbers of dental implant treatments performed, more patients are suffering from implant-associated inflammatory complications, such as peri-implant mucositis and peri-implantitis, which were also suggested to be the most frequent reason for implant failure with a prevalence of 1% to 47% [1,2].

The incidence of peri-implantitis has been reported to be 18–43.9%, making it the most frequent complication in dental implantology [3]. Despite several surgical and non-surgical treatment modalities, clinical outcomes still remain uncertain and there is a lack of explicit recommendations for peri-implantitis therapy [4]. Therefore, various experimental studies have focused on the etiopathogenesis of the peri-implant infections to gain insights into different therapy alternatives.

In the literature, several models have been described to study peri-implantitis by using dogs, minipigs, and rodents as experimental subjects. The “ligature-induced” periodontitis model in rats described by Rovin et al. has been commonly used to initiate peri-implantitis in dogs and murine models [5,6,7]. However, it has been proclaimed that traumatizing periodontal tissues with ligatures is only limited to describe pathological mechanisms of peri-implantitis, and key factors such as the immune system and content of the bio-film could be overlooked [8]. Therefore, topical administration of bacteria has been widely used to initiate periodontal diseases and later peri-implantitis in various animal models in recent years [9,10].

Sun et al. suggested that the *in-vitro* biofilm formation process prior to osseointegration does not relate to the human clinical situation; however, a “self-limiting” process existing in the tissues around natural teeth does not occur in peri-implant tissues [11], and the osseo-disintegration secondary to peri-implantitis is a progressive and continual process, in which rather marginal tissues, all osseous structures along the implant surface, were involved [12,13]. Therefore, in addition to the need for understanding the pathogenesis of the peri-implantitis-related bone loss around implants, the osseo-disintegration process warrants further research.

Moreover, considering the need for examining the effects of novel treatment options of decontamination and consequently re-osseointegration processes, experimental models allowing histological and radiological examinations reproducing pathological processes are still desirable. Therefore, the aim of the current study was to establish a reliable osseo-disintegration model initiated with a single microbe in mini-pigs [14].

## 2. Materials and Methods

All experiments were performed according to the guidelines described by the European Animal Welfare Legislation and in accordance with the European Communities Council Directive (2010/63/EU). Ethical approval was obtained from the ethics committee of Christian Albrechts University (V242-58198 (104-11/18) and experiments were performed in compliance with the appropriate ARRIVE guidelines. The study included three female miniature pigs obtained from commercial supplier (Ellegaard Goettingen Minipigs A/S, Dalmose, Denmark) with an average age of 40 months and an average weight of 39 ± 68 kg. The animals were housed in cages with an enriched environment and had free access to standard food and water ad libitum.

### 2.1. Study Design and Protocol for Surface Contamination

A total of 36 titanium dental implants (3.5 mm in diameter, 9.5 mm in length) with a Ti Grade V surface (Friadent^®^/Ankylos^®^, Dentsply Implants, Mannheim, Germany) was inserted into frontal bone (n: 12) and the basis of the corpus mandible (n: 24). Eighteen implants were contaminated via inoculation of *Enterococcus faecalis* for 7 days. On the first day, the implants were infected with 200 mL of sterile Brain Heart Infusion (BHI) and 100 μL of the overnight culture and then incubated at 37 °C (Scientific C24 Incubator Shaker, New Brunswick Scientific, Edison, NJ, USA). After 4 h, the optical density was controlled via spectrophotometer (BioPhotometer 6131, Eppendorf AG, Hamburg, Germany) at 600 nm (OD600), which was set to 0.8. Nutrient solution was exchanged every 24 h with 200 mL of sterile BHI for 6 days. At the end of the seventh day of incubation, the implant surface was controlled by using chromogenic agar (chromID^®^ VRE, bioMérieux, Nurtingen, Germany) to detect the colored colonies.

### 2.2. Surgical Procedure

**Figure 1:**
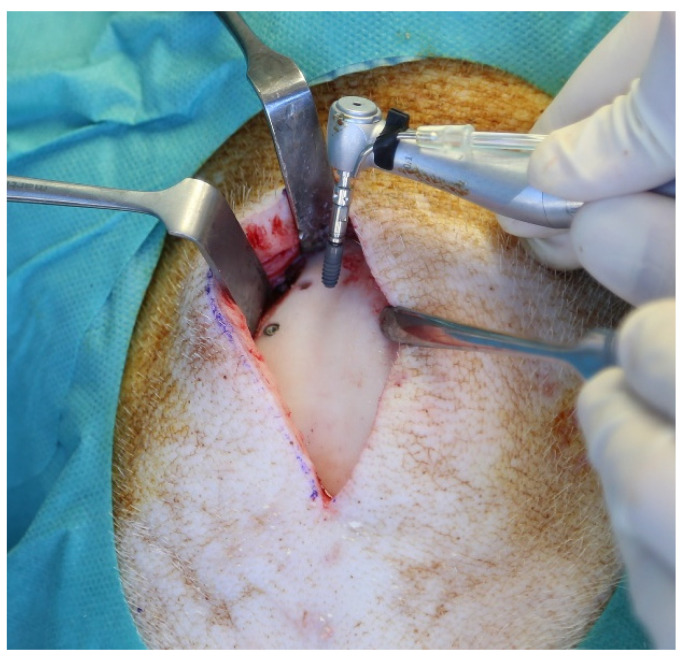
Placement of the implants into the frontal bone.

**Figure 2:**
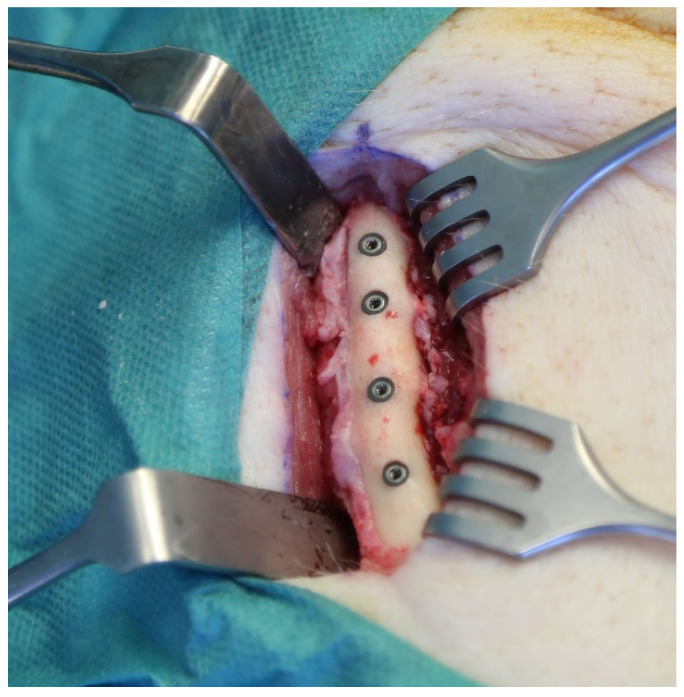
Placement of the implants into the basis of the corpus mandible in the cranial direction.

Implant insertions were performed by a single surgeon with the assistance of an experienced team. Sedation was performed with intramuscular administration of ketamine (10 mg/kg) and midazolam (1.5 mg/kg) injection. Anesthesia was maintained with intravenous ketamine administration. The animals were brought into a prone position in order to assure an appropriate position to operate in the frontal bone area. A total of 6 mL of Articaine HCl 4% was applied transcutaneously at the pre-determined implant recipient site circumferentially. A full-thickness skin incision of 8 cm was selected at the corresponding area and the calvarium was exposed via subperiosteal sharp dissection. Above the fronto-parietal suture, the implant bed was prepared for implant insertion, according to the manufacturer’s guidelines, using the complete sequence of drills for each individual implant with a peak insertion torque of ≥30 N/cm. The distance between the neighboring implants was set to 10 mm. In each animal, two sterile and two contaminated implants were placed bilaterally into the frontal bone, one contaminated and one sterile on each site. (Figure 1). Following the implant placement, a double-layer skin closure was performed. With the same incision and insertion technique, four sterile and four contaminated implants were placed into the basis of the ramus mandible in the cranial direction for each animal containing two sterile and two contaminated implants in each quadrant (Figure 2).

In the postoperative period, metamizole (500 mg, 2 × 1, for 5 days) and ceftriaxone (125 mg/d for 5 days) were administered intramuscularly. To assess the bone regeneration following, fluo-chromes were injected intraperitoneal every 10 days under neuroleptanalgesia:

- day 10: Xylenol-Orange (dosage 90 mg/kg bodyweight, solution 45 mg/mL)
- day 20: Calcein-Green (dosage 15 mg/kg bodyweight, solution 10 mg/mL)
- day 30: Alizarin-Complex (dosage 30 mg/kg bodyweight, solution 15 mg/mL)
- day 40: Tetracycline (dosage 30 mg/kg bodyweight, solution 15 mg/mL)

On the 45th postoperative day, the animals were sacrificed and adjacent tissues were removed and prepared for further examinations.

### 2.3. Sample Processing

Dehydration of formalin-fixed specimens was performed by using ascending concentrations of alcohol tank (Pool of Scientific Instruments, Type 1.42.00, PSI Grünewald, Laudenbach, Germany) followed by embedding in methyl-methacrylate (Sigma Aldrich, St. Louis, MO, USA). The samples were cut parallel to the longitudinal axis of the implants with a diamond-coated grinder (Cut-grinder primus Diamant, Walter Messner, Oststeinbek, Germany). A total of four 40-mm-thick, non-decalcified specimens were obtained for each implant.

### 2.4. Bone-to-Implant Contact, Interthread Bone Density, Peri-Implant Bone Density

The determination of the bone-to-implant contact (BIC) ratio was conducted via quantification of the length of the bone-to-implant contact in relation to the implant perimeter. The measurement started at the mesial shoulder of the implant. To assess the inter-thread bone density (ITBD), the total area of bony structures inside the threads in relation to the inter-thread area within the five most central threads on both sides (mesial and distal) was calculated. The peri-implant bone density (PIBD) was determined by calculating the bone area around the implants in relation to the peri-implanter total area up to a lateral distance of 1 mm. The calculation was conducted at the five most central threads on the distal and mesial sides. The assessment was performed by a single researcher, who was blinded to the study regarding the subgroups and study design. Histological examinations were conducted with toluidine blue staining. Fluorescence microscopy (Axio Oberserver.Z1 and Axio Cam and Axio Vision, Carl Zeiss Mikroskopie, Jena, Germany) and light microscopical examinations were also performed. For the digital analysis of histologic specimens, a Leica QWin Standard (V 3.2.0 Leica Microsystems Imaging Solution, Cambridge, UK) system was used.

### 2.5. Microradiography

The implants with the surrounding bone were taken and put on a high-resolution microradiography plate with a resolution of 2000 lines/mm (High Resolution plates (Kodak^®^, Rochester, NY 14650, USA). Afterwards, exposure was performed in a Faxitron 53855A (Hewlett -Packard, McMinnville, OR 97128, USA) at a focus distance of 16 cm at 25 kV and 3 mAs for 390 s for 70 µ samples and up to 630 s for 110 µ thickness, respectively. A Kodak^®^ HRP developer was used for embedding the samples (for 5 min at 20 °C) under constant movement swing (HRP developers/distilled water: ratio of 1/3).

The development in 1% acetic acid bath was paused after 60 s. Fixation was conducted for 600 s with agitation (Kodak^®^ fixer 3000A/distilled water: 1/3) followed by washing for 900 s with distilled water. For final rinsing, Agepon^®^ (400 mL of distilled water and 2 mL Agepon^®^) was used for 60 s. The plates were air-dried followed by covering with 4 × 4.5-cm glasses using n—butyl acetate and Eukitt-air application for 1 day. An air extractor was used to maintain a drying period of 1 week. After that, the samples were digitized under a light microscope at a magnification of 1:18. Adobe Photoshop 7.0 for Windows was used to evaluate the digital data.

### 2.6. Statistical Analysis

A power analysis was carried out by using the paired t test power calculation to calculate the sample sizes per group. A statistical significance with a power of 80% was recognized. Data sets were tested for distribution normality and homogeneous variance. Two-way analysis of variance (ANOVA) followed by the Tukey HSD post hoc test (SPSS Statistics 20.0, IBM Corporation, Armonk, NY, USA) was used for comparative assessment. The significance level was set to a *p* value of 0.05.

## 3. Results

All implants were successfully contaminated with *Enterococcus faecalis.* The contamination of the implants with *Enterococcus faecalis* led to an explicit degradation of bone growth at the implant-surrounding area. Almost no bone contact to the implant was achieved. Except for the lack of osseointegration at the contaminated surfaces, there were no signs of inflammation in the adjacent hard and connective tissues, no wound healing disturbances, and no systematic interference or indispositions shown by the animals’ behavior.

### 3.1. Histomorphometric Analysis

Comparative assessment of BIC revealed that, compared to the sterile implants, contaminated implants showed significantly reduced values (*p* < 0.001), irrespective of the placement. According to the ITBD values, sterile implants showed superior interthread bone density (61.3 ± 4.4%) compared to the infected implants (8.7 ± 3.4 %, *p* < 0.001). The PBD values were also significantly higher around the sterile implants, 80.8 ± 2.4 %, compared to the contaminated ones, 43.9 ± 5.0%. (*p* < 0.001) (Table 1).

Regarding the implant recipient sites, no differences could be observed in terms of BIC, ITBD, and PBD (Table 2).

**Figure 3:**
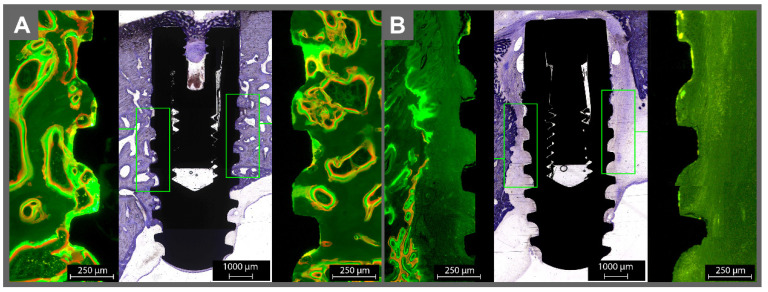
The histological examination of the (**A**) sterile and (**B**) contaminated implant in the frontal bone. Please note the lack of osseointegration around the contaminated implant. Around the sterile implant, the green and red fluorophores were overlapping and surrounding the implant without gaps, indicating healthy bone growth on the implant surface. Orange fluorescence was found on bone trabecula. Around the infected implants, almost no fluorescence was found on the surface, except in some areas with a very thin line of green fluorescence.

**Figure 4:**
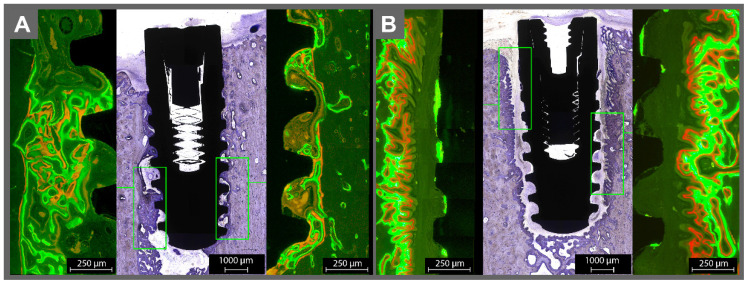
The histological examination of the (**A**) sterile and (**B**) contaminated implant into the basis mandible. Please note the lack of osseointegration around the contaminated implant. Around the sterile implants, the green and red fluorophores were overlapping and surrounding the implant without gaps, indicating healthy bone growth on the implant surface. Orange fluorescence was found on bone trabecula. On adjacent bone to the infected implants, almost no fluorescence was found on the implant surface, except in some areas with a very thin line of green fluorescence.

Around the sterile implants, the green and red fluorophores were overlapping and surrounding the implant without gaps, indicating healthy bone growth on the implant surface (Figure 3A and Figure 4A). Orange fluorescence was found on bone trabecula (given at day 10, representing the remodeling phase after resorption of the “damaged” bone due to the implantation). For the infected implants, almost no fluorescence was found on the contaminated surfaces, except in some areas with a very thin line of green fluorescence on the implant surface (Figure 3B and Figure 4B). The implants were surrounded by connective tissue. The bone adjacent to the connective tissue showed fluorescence activity in green and red.

With the help of the toluidine blue staining, the newly accumulated bone could be detected, which appeared slightly darker than the mature bone. Around the sterile implants, new bone stood in direct contact with the implant surface, coronal area on the sealing cap, and bone surface. The drilling canal was detectable and underwent remodeling. The contaminated implants had almost no bone contact. The drilling canal was not detectable. Even more, it seemed as if extensive bone resorption took place until a sufficient gap to the contamination was reached. Nevertheless, newly formed (darker) apposition of the new bone, which seemed like it was growing towards the implant, could be observed.

### 3.2. Micro-Radiographic Analysis

**Figure 5:**
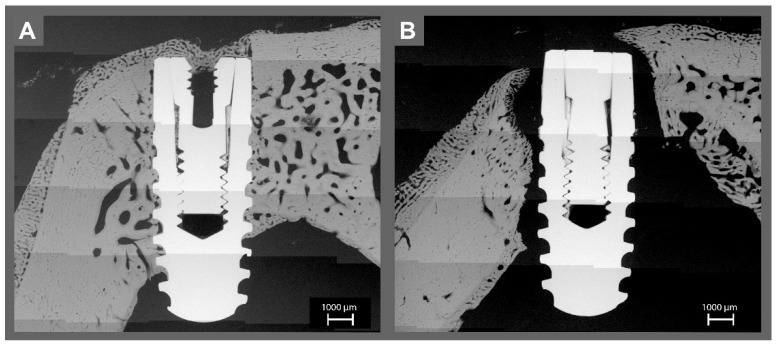
The micro-radiographic examination of the corresponding (**A**) sterile and (**B**) contaminated implants placed into the frontal bone. Please note the lack of osseointegration around the contaminated implant.

**Figure 6:**
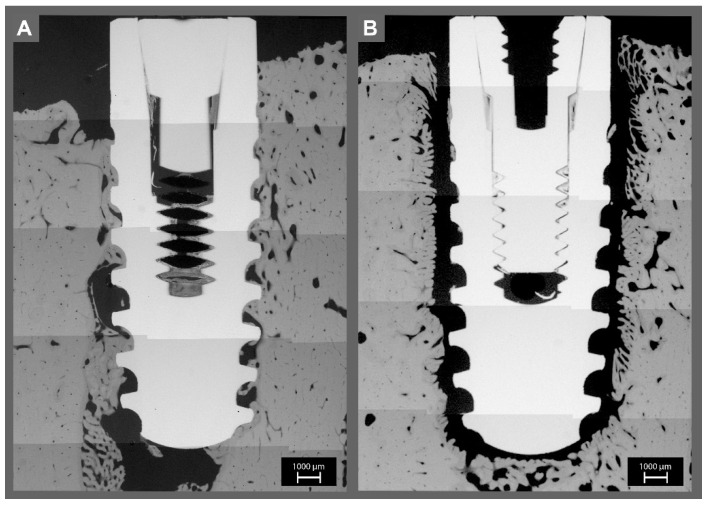
The radiological examination of the same samples of (**A**) sterile and (**B**) contaminated implants inserted into the basis mandible. Please note the lack of osseointegration around the contaminated implant.

Micro-radiographic analysis showed apparently the lack of osseointegration around contaminated implants. The bone density around all implants appeared similar, with a slight contrast difference between spongiosa and cortical bone (Figure 5 and Figure 6).

## 4. Discussion

The aim of this study was to investigate the healing, remodeling, and bone response when confronted with infected implants via a novel osseo-disintegration model in minipigs. The bone of minipigs resembles the human bone in anatomy, morphology, healing, remodeling, mineral density, and bone mineral concentration. The pigs lamellar bone structure is comparable to human bone, except for a denser trabecular network [15,16,17]. It was suggested that the minipigs’ bone micro- and macrostructure is suitable to model the human implant-healing processes [17,18].

The selected implant recipient sites were the forehead and the basis of the lower jaw. The foreheads’ behalf was a convenient approach; the disadvantage was given by the extensive sinus and, therefore, narrow bone volume. The mandibular basis presented a sufficient amount of bone volume. As the implants were not placed in the oral cavity, the freshly operated side was not stressed by the masticatory movement. In the current experimental model, the quantification of the healing process and the connections between implants and bone were assessed via BIC, ITBD, and PBD. Those parameters were used as markers for osseointegration in several studies [19,20]. According to Bernhardt et al., these parameters could appropriately describe the osteogenic potential of the implant surfaces [21].

BIC values for sterile implants measured 6 weeks after placement (58.5 ± 5.2%) were similar to those reported by Fabbro et al. [17], where a BIC of 80.79 ± 5.61% was reported for SLA implants after 12 weeks. Similarly, Cochran et al. [22] found BIC values of 68% after 4 weeks and 79% after 8 weeks of insertion. Fabbro et al. reported the highest BIC values after a healing period of 3 months [17]. In contrast, the BIC of contaminated implants was significantly reduced to an average of 11.5 ± 2.0%, demonstrating how effectively bacteria engage in the healing process.

Because peri-implantitis has become a topic of interest, several approaches were presented to mimic the bacterial colonization of surfaces [23,24]. Recent studies have used mixed microbial infection by oral lavage to facilitate bacterial adherence to the implant surface and to induce an inflammatory host response in animal models [10]. In the current experimental research, *Enterococcus faecalis* was used for decontamination. An animal model resembling the multi-colonial human bacterial infection may improve the understanding of the issue and help to identify appropriate treatment schemes.

A different approach to verify the bacterial occurrence is the use of plates or flosses, which are fixed in the oral cavity over different time spans [25]. All these experiments provide the proof of the complexity of the bacterial colonization at a sterile outset, contrary to our study, where the implants were already infected at the moment of insertion. Sousa et al. used a similar approach, where sandblasted, large-grit, acid-etched titan discs were infected with a biofilm and placed in the tibia of rabbits [26].

The micro radiography, fluorescence microscopy, and toluidine staining consistently showed that the contaminated implants lacked direct bone contact. Instead, soft tissue was found to cover the implant. The soft tissue generated a kind of barrier to the surrounding bone, not unlike reactive scar tissue. There was only a very slight evidence of contact osteogenesis on the contaminated surfaces, which may have originated from a loss of bacteria during the insertion of the implant in the bone; thus, the contaminated implants induced a distance osteogenesis, where bone growth occurs from the surrounding established bone, instead of contact osteogenesis, where the implant surface is directly connected to the bone. Further studies with long-term follow-up periods would be beneficial to determine the osseointegration process. Preeminent is also the lack of inflammation signs like pus, edema, erythema, abscess-forming, deficient or complete absence of wound healing, or any systemic reactions. The important outcome of this study is that the bacteria appeared only to affect the direct implant bone contact and were not, as one should expect, leading to systemic side effects.

Previous authors have suggested that plaque accumulation is necessary for bone resorption to occur in response to a ligature-induced peri-implantitis model; but, Baron et al. [27] showed the lack of validation for this claim in their review and concluded that other factors, such as a foreign body reaction to the ligature, may also trigger a peri-implant inflammatory response [28]. Therefore, a closed system instead of a ligature-induced peri-implantitis model was used in the current experimental model. Additionally, a mono-infection model was selected to obtain a standardized biofilm formation.

Despite several studies that defined *Enterococcus faecalis* as a “key stone” player in peri-implantary bone loss, it is obvious that the *Enterococcus faecalis* is not the predominant infectious agent in peri-implantitis. In the current study, *Enterococcus faecalis* was selected due to its simplicity in isolation and its suitability in biofilm formation in *ex-vivo* studies [29].

A recent study focusing on the histological aspects of peri-implantitis [12] also revealed that not only supracrestally but also adjacent to whole threads of the affected implant caudally, where the bone resorption macroscopically did not appear, an increased concentration of osteoclasts was present. Therefore, the whole surface of the implant was contaminated with *Enterococcus faecalis* in the model described herein.

## 5. Conclusions

There is an ongoing need for feasible experimental models in peri-implantitis research due to the growing number of novel therapeutic approaches such as photodynamic therapy [30] or implant surface modifications [31,32]. Despite its limitation regarding the lack of mimicking the multi-bacterial characteristics of the oral flora, the current experimental model could be an appropriate option to realize a significant alteration of dental-implant osseointegration and allow the examination of novel surface decontamination techniques without impairing local and systemic inflammatory complications.

## Figures and Tables

**Table 1:** The histomorphometric parameters are shown in Table 1. Compared to the sterile implants, the contaminated implants showed significantly reduced BIC values (*p* < 0.001), irrespective of the placement. ITBD showed similar results: Sterile implants showed an interthread bone density of 61.3 ± 4.4% compared to the infected implants with an ITBD of 8.7 ± 3.4 % (*p* < 0.001). The PBD values were higher around the sterile specimens, 80.8 ± 2.4 %, compared to contaminated ones, 43.9 ± 5.0% (*p* < 0.001).

	Sterile	Contaminated	*p*-Value
BIC [%]	58.8 ± 3.4	7.61 ± 2.2	*p* < 0.001
ITBD [%]	61.4 ± 4.4	8.7 ± 3.4	*p* < 0.001
PBD [%]	78.6 ± 2.4	40.6 ± 2.9	*p* < 0.001

**Table 2:** Comparative assessment of BIC, ITBD, and PBD values in both groups showed no significant differences between implants placed in frontal bone and mandible.

	**Fb Sterile**	**Fb Contaminated**	***p*-Value**
BIC [%]	64.2 ± 2.4	8.2 ± 5.2	*p* < 0.001
ITBD [%]	73.6 ± 6.7	0.8 ± 0.7	*p* < 0.001
PBD [%]	63.9 ± 5.9	22.8 ± 4.5	*p* < 0.001
	**M Sterile**	**M Contaminated**	***p*-Value**
BIC [%]	56.2 ± 4.7	7.2 ± 1.7	*p* < 0.001
ITBD [%]	53.2 ± 4.9	14.0 ± 5.2	*p* < 0.001
PBD [%]	86.0 ± 1.5	54.2 ± 3.1	*p* < 0.001
	**Frontal Bone**	**Mandible**	***p*-Value**
BIC [%]	30.01 ± 4.2	29.03 ± 2.7	*p* > 0.001
ITBD [%]	34.9 ± 4.7	35.48 ± 1.4	*p* > 0.001
PBD [%]	61.82 ± 2.4	62.0 ± 4.6	*p* >0.001

## Data Availability

All data are available in the archive of the Research Laboratories, Christian Albrechts University, Department of Oral and Maxillofacial Surgery.
